# Retraction: A non-catalytic function of carbonic anhydrase IX contributes to the glycolytic phenotype and pH regulation in human breast cancer cells

**DOI:** 10.1042/BCJ20190177_RET

**Published:** 2026-06-03

**Authors:** Susan Frost, Mam Y. Mboge, Zhijuan Chen, Daniel Khokhar, Alyssa Wolff, Lingbao Ai, Coy D. Heldermon, Murat Bozdag, Fabrizio Carta, Claudiu T. Supuran, Kevin D. Brown, Robert McKenna, Christopher J. Frost

**Affiliations:** Department of Biochemistry and Molecular Biology, University of Florida, 1200 Newell Dr, Gainesville, FL 32610, U.S.A.

**Keywords:** breast cancers, carbonic anhydrase IX, glycolysis, MCT4, microenvironment, pH control

This article is being retracted from the *Biochemical Journal* at the request of the Editorial Board and its Chair. This action follows the receipt of a notification by the journal *PLOS One*, alerting the Editorial Board to similarities between figures in this article and another article by the same author group (Mboge et al. (2018) DOI: 10.1371/journal.pone.0207417).

Upon investigation, a similarity was also identified by the Editorial Office between two figures within this article. The similarities specifically involve the EV blots from Figure 1B T47D/CAXII and Figure 4A T47D/CAXII being identical despite being associated with different loading controls as presented in the paper, as well as with the Figure 2B T47D/CAXII blots from Mboge et al. 2018.

The authors fully cooperated with our investigation and provided repeated data. Although the authors were not able to provide the original data or to explain the image duplication within and across papers, they were able to replicate the results under the same experimental conditions and maintain that the conclusions of the work are valid. However, they have been unable to provide original raw data for the requested western blots.

In the absence of the original raw data and given that the duplication also occurs across two different articles, the Editorial Board feels that the appropriate course of action is to retract the article. The authors disagree with this decision.

